# Response of foxtail millet yield, soil chemical property and bacterial community to different green manure-foxtail millet rotation models in North China

**DOI:** 10.3389/fmicb.2025.1558354

**Published:** 2025-03-21

**Authors:** Guohong Yu, Ya Han, Pengcheng Liu, Hongbo Hao, Mingzhe Li

**Affiliations:** ^1^Key Lab of Crop Drought Tolerance Research of Hebei Province, Institute of Dry Farming, Hebei Academy of Agriculture and Forestry Sciences, Hengshui, China; ^2^Hebei Center for Ecological and Environmental Geology Research, Hebei GEO University, Shijiazhuang, China

**Keywords:** green manure-foxtail millet rotation, soil physicochemical properties, bacterial community, high-throughput sequencing, foxtail millet yield

## Abstract

China is a largely agricultural country, while the drought climate in northern of China is more and more severe, which influences on the agriculture production seriously. The over-exploitation of groundwater is a critical issue in the low plains of Hebei Province. To address this challenge, the government has implemented winter fallow and rain-fed crop planting policies. In alignment with these policies and ensure the sustainable utilization and protection of cultivated land, this study conducted long-term field experiments using three green manure with foxtail millet rotation models at Shenzhou District experimental base, Hengshui City, Hebei Province. Thefoxtail millet yield, soil bacterial community characteristicsc, and soil physicochemical properties were analyzed to identify an optimal green planting model for promoting sustainable agricultural development. The results revealed that three green manure-foxtail millet rotation models significantly increased millet yield compared to millet-rallow rotation. The foxtail millet–Triticum secale rotation model achieved the highest yield increase, with a 12.47% average improvement in thousand-seed weight in 2021 and 2022 compared to millet-fallow rotation. This rotation model also led to the largest increase in available phosphorus content, which rose by 46.16 and 37.56% in 2021 and 2022, respectively. Furthermore, the diversity and richness of the soil rhizosphere bacterial community were highest under this model. Beneficial bacterial genera, including those in the Phyla Bacteroidetes and Proteobacteria, were more abundant, while the relative abundance of Acidobacteria was lowest. Correlation analysis showed that soil organic matter, available phosphorus, and millet yield were positively correlated with multiple genera of *Bacteroidetes* and *Proteobacteria* but negatively correlated with *Acidobacteria*. In conclusion, the foxtail millet–*Triticum secale* rotation model effectively improved the soil environment and supported stable, high millet yields. These findings provide a theoretical basis for advancing crop rotation strategies and offer technical support for sustainable agricultural development.

## 1 Introduction

China is a largely agricultural country, while the drought climate in northern of China is more and more severe, which influences on the agriculture production seriously (Li et al., 2023). The over-exploitation of groundwater is a critical issue in the low plains of Hebei Province. In alignment with these policies and ensure the sustainable utilization and protection of cultivated land, the crop types that use water efficiently and planting patterns were in urgent demand. However, Foxtail millet (*Setaria italica*) is a short-season, drought and barren-tolerant crop, making it highly suitable for cultivation in arid and mountainous regions across northern and southern China (Nielsen and Vigil, 2017). Given the increasing global challenges posed by drought, dry farming systems have garnered significant attention (Diao and Cheng, 2017). Due to the millet having characteristics of rich nutrition, the balance of various components, excellent storage properties, making it an ideal crop for addressing dietary nutritional needs and serving as a strategic reserve in water-scarce scenarios (Zhang et al., 2018). These unique attributes have contributed to the rising demand and development of the millet industry. However, continuous foxtail millet-rallow rotation to achieve high yields has led to significant challenges, including reduced crop productivity, severe soil borne diseases, and declining soil quality (Zhang et al., 2024). Crop rotation offers an effective strategy to mitigate the adverse effects of continuous cropping (Anurag et al., 1997; Sammauria et al., 2020). Reasonable crop rotations can balance soil nutrient levels, improve soil physicochemical properties, regulate microbial communities, and enhance crop yields (Horn and Alweendo, 2011). For instance, millet rotations with crops such as pigeon pea or *Urochloa* have been shown to reduce soil pH and aluminum levels while increasing calcium, magnesium, potassium, and iron content. These rotations also improve soil structure and physical properties relative to fallow systems (Nascente and Stone, 2018). Furthermore, millet rotations promote complex bacterial community networks abundantly, enriching taxa such as *Actinobacteria*, *Chloroflexi*, and *Proteobacteria* (Li et al., 2024). Despite these benefits, how specific green manure-millet rotations affect soil properties and bacterial communities is unclear.

Green manure is a vital agricultural management measure that enhances soil fertility, accelerates nutrient cycling, and improves crop yields when returned to the soil (Zhang et al., 2017; Yang et al., 2023). Green manure crops, including grasses and legumes, influence the availability of carbon and nitrogen, with legumes fixing atmospheric nitrogen and grasses contributing higher carbon levels (Khan et al., 2020). These differences also influence on soil microbial communities, further improving soil quality (Chavarría et al., 2016; Xu et al., 2023). Despite of so many advantages, the adoption of green manure remains limited. Expanding the utilization of green manure in rotation systems is a promising strategy for increasing crop yields and preserving environmental health (Li et al., 2020). Investigating the effects of green manure-millet rotations on soil chemical and biological properties is particularly relevant in northern China.

Soil microorganisms are crucial for driving biogeochemical cycles in agroecosystems (Xia et al., 2023). Among these, soil bacteria play significant role in decomposing green manure residues, cycling nutrients, and ultimately react on the crop productivity (Leite et al., 2021). Recent studies have demonstrated strong correlations between specific bacterial taxa and soil properties, positioning bacterial communities as valuable indicators of soil quality across ecosystems (Trivedi et al., 2016; Qian et al., 2022). The research has shown that milk vetch as green manure alters bacterial communities in paddy soils and significantly correlates with soil pH in rice-green manure systems (Yang et al., 2023; Gao et al., 2021). Additionally, rapeseed manure has been shown to enhance soil nutrients, enzyme activities, bacterial diversity, and the prevalence of beneficial bacteria (Li et al., 2019). However, the mechanisms underlying changes in soil quality and bacterial communities under different green manure-millet rotations remain largely unexplored. To address these gaps, we conducted a five-year field experiment to evaluate the effects of four green manure-millet rotation systems (fallow-millet, *Triticum secale*-millet, *Brassica napus L.*-millet, and *Orychophragmus violaceus*-millet) on millet yield, soil chemical properties, and soil bacterial communities. The content of this study were to: (1) Assess the impact of green manure-millet rotation systems on millet yield and soil chemical properties. (2) Investigate how soil bacterial community composition responds to different rotation systems. (3) Explore the relationships between soil bacterial communities, chemical properties, and millet yield across different rotations.

## 2 Materials and methods

### 2.1 Site description and experimental design

The green manure-foxtail millet rotation experiment was conducted in Hujiachi Town (37°44′N, 115°47′E), Hengshui City, Hebei Province, North China. Established in 2017. The experiment site is characterized by a warm temperate continental monsoon climate, with an annual mean temperature of 12.4°C and an average annual precipitation of 550 mm, predominantly occurring from June to September. The soil at the site is classified as loam fluvo-aquic soil. At the start of the experiment, the chemical properties of the topsoil (0–20 cm) were as follows: organic matter (OM) content of 20.0 g kg^−1^, alkali-hydrolyzable nitrogen (AN) of 70.66 mg kg^−1^, available phosphorus (AP) of 33.81 mg kg^−1^, and available potassium (AK) of 190.52 mg kg^−1^.

The experiment followed a randomized block design with three replicate plots per treatment. Four rotation treatments were implemented: (1) Fallow-millet rotation (Si-Le) (Control), (2) *Triticum secale*-millet rotation (Si-Ts), (3) *Brassica napus L.*-millet rotation (Si-Bn), (4) *Orychophragmus violaceus*-millet rotation (Si-Ov) For winter green manure, Triticum secale, *Brassica napus L.*, and *Orychophragmus violaceus* were utilized. Millet was sown in late June and harvested in late September. The cultivars used in the study were as follows: *Henggu 13* for millet, *Zhongai 1048* for *Triticum secale*, *Hengyou 8* for *Brassica napus L.*, and a conventional *Orychophragmus violaceus* variety. Millet planting adopt the strategy of sowing after rain and not watering the whole growth period, using compound fertilizer (25 kg/acre) as the base fertilizer. The long-term experiment spanned five consecutive years (2018–2022), with consistent field management practices across all plots except for the variation in winter crop species used in the rotations.

### 2.2 Soil sampling and millet harvest

After the millet ripened, grain yields were determined by a single harvest from each plot. Yield components, including ear diameter, panicle weight, grain weight, and thousand-seed weight, were measured in the laboratory. The yield of millet (kg/hm^2^) were converted by measuring the millet yield of 100 m^2^. Following the millet harvest in 2021 and 2022, soil samples were collected from the top 0–20 cm layer for physiochemical property analysis. Five soil cores were taken from each plot and combined into a single composite sample. In September 2021, a total of 20 composite soil samples (four treatments × five replicates) were collected. Collected the plants (Z-shaped sampling method), removed the bulk soil at the root, placed them in a foam box filled with dry ice, in a dark environment, and returned to the laboratory as soon as possible; In a sterile workbench, shaked the plant roots, removed the loose soil in the roots, and collected the residual soil from the roots with a sterile brush for further analysis. Each soil sample was divided into two subsamples: one was passed through a 2-mm sieve and air-dried for physiochemical property analysis, while the other was stored at −80°C for DNA extraction.

### 2.3 Soil chemical property analysis

Soil pH was measured using a pH meter (soil-to-water ratio of 1:2.5) and the electrical conductivity (EC) was evaluated using electrode method. Soil organic matter (OM) was determined by potassium dichromate oxidation with external heating (Shen et al., 2021). Soil alkali-hydrolyzed nitrogen (AN) was determined using alkaline hydrolysis diffusion method (Cheng et al., 2023 Wan et al., 2024). The available phosphorus (AP) was determined using 0.5 mol L^−1^ NaHCO_3_ extraction-molybdenum antimony anticolorimetric method (Zhu et al., 2021). The available potassium (AK) is determined by flame photometry (Fu et al., 2020).

### 2.4 Soil microbial DNA extraction and illumina NovaSeq sequencing

Microbial DNA was extracted from 0.5 g of soil using a genome extraction kit (DP336, Beijing Tiagen Biotechnology Co., Ltd.). DNA quality was assessed using 1% agarose gel electrophoresis. Specific primers with barcodes were designed for the designated sequencing region. PCR products were amplified, detected by agarose gel electrophoresis, and purified using the Agencourt AMPure XP nucleic acid purification kit. Denatured sodium hydroxide was used to produce single-stranded DNA fragments, which were sequenced using the Illumina MiSeq platform (Allwegene technology Co., Ltd., Beijing). The V3-4 hypervariable region of bacterial 16S rRNA gene were amplified with the universal primer 338F (*5*′-ACTCCTACGGGAGGCAGCAG-*3′*) and 806R (*5′*-GGACTACNNGGGTATCTAAT-*3′*). The quality control was performed on the raw sequencing data to generate optimized sequences. Operational taxonomic unit (OTU) clustering and annotation were subsequently conducted (Rognes et al., 2016). Alpha and beta diversity analyses were performed to assess bacterial diversity (Amato et al., 2013; Wang L. et al., 2022; Wang X. Y. et al., 2022). Annotation results provided classification information at all levels, allowing for correlation analysis of sample composition and differences in community structure (Segata et al., 2011). Sequencing data are available in the Sequence Read Archive (SRA) database at NCBI (accession number: PRJNA733689).

### 2.5 Statistics

One-way analysis of variance (ANOVA) was performed using SPSS 21 software to evaluate differences in yield components, soil chemical properties, and bacterial relative abundance across rotation models. Tukey’s test was applied for multiple comparisons at a significance level of *p* < 0.05. Principal component analysis (PCA) was used to analyze the beta diversity of bacterial communities. Heat maps illustrating bacterial abundance at different genus levels were generated using Origin 2022. Redundancy analysis (RDA) was conducted with CANOCO 5.0 software to evaluate correlations between soil chemical properties and bacterial genera (Zeng and An, 2021). Phylogenetic tree was constructed using the nearest neighbor association method in MEGA 6.0 software to analyze bacterial colony characteristics (Wei et al., 2020). Data processing and visualization were performed using Microsoft Excel 2010 and DPS 7.05 software.

## 3 Results

### 3.1 The variation of millet yield

**Figure 1:**
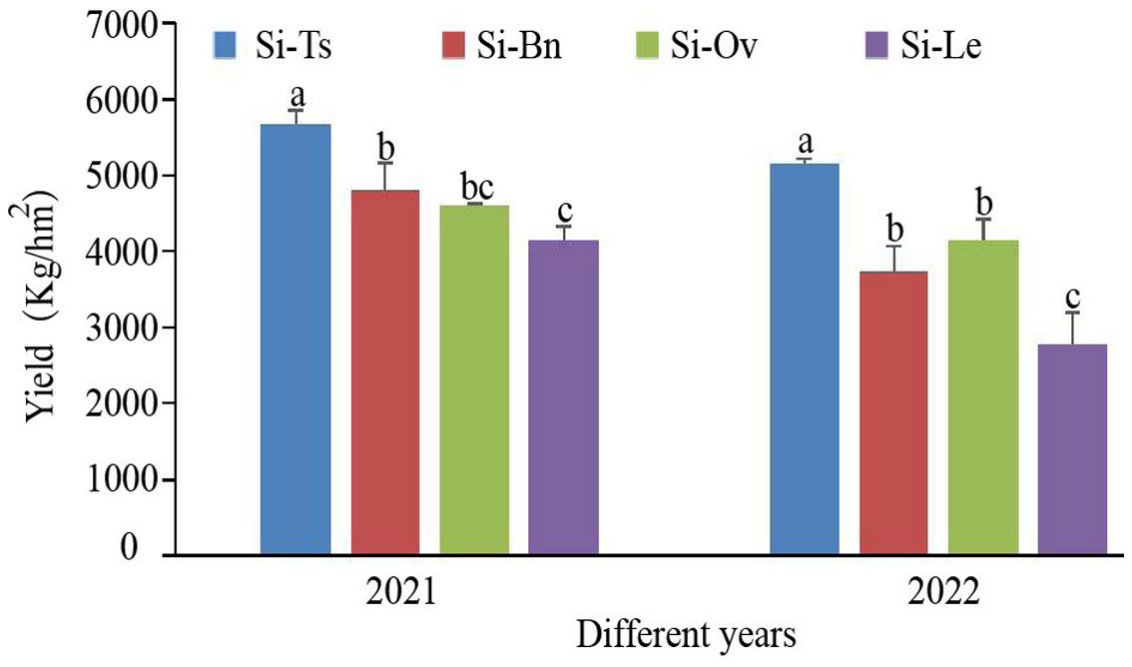
The millet yield of different rotation patterns in 2021 and 2022.

The millet yields in three manure-rotation patterns are significantly different from that in Si-Le in 2021 and 2022. In 2021, the grain yield of millet followed the order Si-Ts > Si-Bn > Si-Ov > Si-Le, and the grain yield of Si-Ts was the highest (5676.45 kg-hm^−2^), which was 36.72% higher than that of Si-Le, and that of Si-Bn was 15.63% higher than that of Si-Le. In 2022, the yield of millet was Si-Ts > Si-Ov > Si-Bn > Si-Le, and the yield of Si-Ts was the highest (5152.34 kg. hm^−2^), which was significantly increased by 85.67% compared with that of Si-Le. Additionally, The yield of Si-Bn and Si-Ov was 34.77% and 49. 56% obviously higher than that of Si-Le (Figure 1).

Different rotation patterns have different effects on millet yield components (Table 1). In 2021, compared with Si-Le, Si-Ts significantly increased ear diameter and 1,000-grain weight by 5.00% and 10.60%, respectively. The grain yield of Si-Bn was 3.91% higher than that of Si-Le. Compared with Si-Le, Si-Ov significantly increased panicle weight, grain weight per spike and The output rate of the valley by 13.72, 20.35 and 5.82%, respectively. In 2022, compared with Si-Le, the spike weight, spike grain weight and 1,000-grain weight of Si-Ts rotation were significantly increased by 17.06%, 39. 41, and 14.34%, respectively. Si-Ov rotation significantly increased spike weight and spike grain weight by 35.88 and 20.46%, respectively.

**Table 1:** Effects of different rotation patterns on components of millet yield.

Year	Crop rotation mode	Ear diameter (cm)	Panicle weight (g)	Grain weight per spike (g)	The output rate of the valley (%)	Thousand grain weight (g)
2021	Si-Ts	2.11 ± 0.13a	20.31 ± 1.51ab	15.64 ± 0.71ab	77.01 ± 1.00c	3.34 ± 0.05a
	Si-Bn	2.04 ± 0.15b	18.72 ± 0.30b	15.01 ± 1.00b	80.22 ± 1.08b	3. 0 ± 0.10ab
	Si-Ov	2.03 ± 0.20b	21.64 ± 1.35a	17.68 ± 1.04a	81.69 ± 1.53a	3. 4 ± 0.05ab
	Si-Le	2.01 ± 0.11b	19.03 ± 1.72b	14.69 ± 1.13b	77.20 ± 1.64c	3. 2 ± 0.23b
2022	Si-Ts	2.04 ± 0.13a	15.92 ± 1.21b	15.60 ± 0.53a	84.65 ± 0.90a	3.03 ± 0.24a
	Si-Bn	2.10 ± 0.10a	13.48 ± 1.35c	11.11 ± 0.85c	82.47 ± 1.67a	2.88 ± 0.09ab
	Si-Ov	2.10 ± 0.08a	18.48 ± 0.72a	13.48 ± 0.03b	84.37 ± 1.30a	2.87 ± 0.18ab
	Si-Le	2.02 ± 0.10a	13.60 ± 0.36c	11.19 ± 0.66c	82.42 ± 1.43a	2.65 ± 0.06b

Different letters are significantly different at *p* < 0.05.

### 3.2 The changes of soil chemical properties

After 5 years of continuous planting, the soil physical and chemical properties varied significantly in different rotation patterns (Table 2). In 2021, compared to the Si-Le control, the organic matter and available phosphorus in Si-Ts and Si-Bn rotation pattern both displayed notable improvements. Organic matter content increased by 17.86 and 16.33%, and available phosphorus level increased significantly by 113.82 and 46.16%, respectively. In 2022, the Si-Ts rotation demonstrated significant enhancements in alkaline nitrogen, available phosphorus, and available phosphorus, with increases of 17.68, 37.56, and 12.56%, respectively, compared to Si-Le. But the millet yield in 2022 experienced a notable decline compared to 2021, with reductions ranging from 9.23 to 33.16%.

**Table 2:** Effects of different rotation patterns on topsoil physical and chemical properties.

Year	Rotation mode	pH	Electrical conductivity (μs. cm^−1^)	Organic matter (g. kg^−1^)	Alkali-hydrolyzed nitrogen (mg. kg^−1^)	Available phosphorus (mg. kg^−1^)	Available potassium (mg. kg^−1^)
2021	Si-Ts	7.98 ± 0.16a	145.97 ± 3.30ab	23.12 ± 0.61a	94.97 ± 0.76a	30.94 ± 1.31a	179.77 ± 2.46b
	Si-Bn	8.10 ± 0.20a	150.40 ± 2.08a	22.80 ± 0.32a	85.29 ± 1.81b	21.15 ± 1.14b	143.59 ± 0.23c
	Si-Ov	8.03 ± 0.20a	142.27 ± 2.08b	20.21 ± 0.73b	86.26 ± 2.06b	16.31 ± 0.92c	139.68 ± 0.01d
	Si-Le	7.92 ± 0.33a	146.97 ± 5.25ab	19.62 ± 0.16b	93.76 ± 0.76a	14.47 ± 1.09c	206.14 ± 1.59a
2022	Si-Ts	8.30 ± 0.25a	128.87 ± 14.39a	20.81 ± 3.23a	89.44 ± 5.50a	18.57 ± 3.68a	135.30 ± 0.07a
	Si-Bn	8.34 ± 0.07a	113.73 ± 6.86a	17.87 ± 2.44a	74.94 ± 9.24b	14.08 ± 1.01b	120.42 ± 3.62b
	Si-Ov	8.26 ± 0.04a	114.40 ± 8.82a	17.94 ± 2.82a	75.74 ± 4.97b	14.21 ± 1.91b	121.60 ± 3.41b
	Si-Le	8.32 ± 0.10a	120.00 ± 9.08a	18.03 ± 0.50a	76.00 ± 4.94b	13.50 ± 1.50b	120.20 ± 4.37b

### 3.3 The difference of bacterial communities

**Figure 2:**
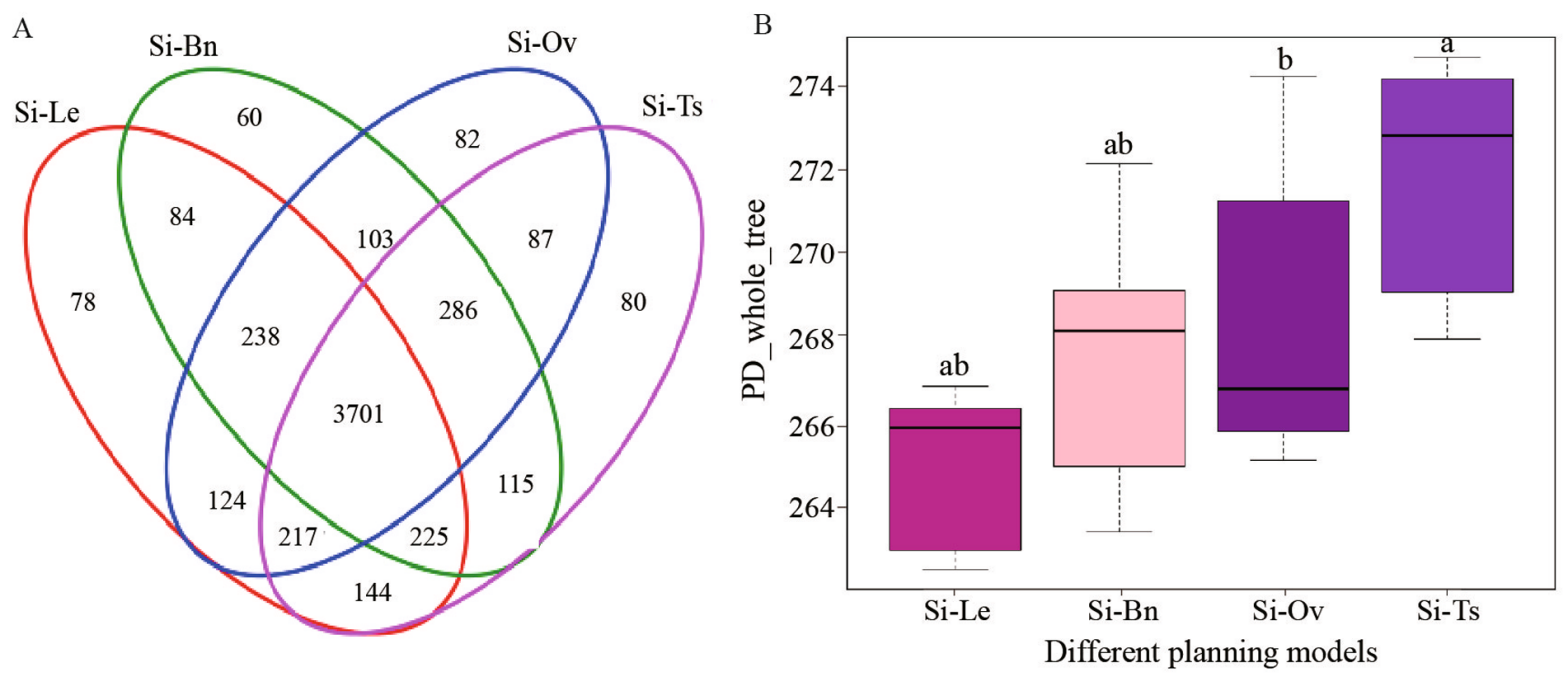
The differential analysis of bacterial communities diversity cross rotation patterns. **(A)** Venn diagram illustrating the differences in OTUs among planting models. Each color represents a different rotation pattern, with the numbers inside each oval indicating the total OTUs unique to that model. Overlapping areas represent common OTUs shared between different planting models. **(B)** Alpha diversity analysis of bacterial communities. The horizontal axes represent different rotation pattern, the vertical axes represent pedigree diversity indexes. The scales of horizontal and vertical axes are relative distance and have no practical significance.

A Venn diagram was used to analyze differences in the operational taxonomic unit (OTU) data for bacterial communities in different rotation patterns, as shown in Figure 2A. The significant differences in the number of OTUs were observed among the four groups, with varying degrees of specific OTUs compared to the millet monoculture (Si-Le). Specifically: 1127 OTU differences were found between the Si-Bn group (millet and *Brassica napus* rotation) and the Si-Le group (millet and fallow rotation). One thousand and eighty-four OTU differences were identified between the Si-Ov group (millet and *Orychophragmus violaceus*) and the Si-Le group. One thousand and ninety-two OTU differences were observed between the Si-Ts group (millet and Triticum secale rotation) and the Si-Le group. Alpha diversity analysis was conducted to assess bacterial community richness within each cropping mode, as illustrated in Figure 2B. The median values of the bacterial community index in the Si-Ts, Si-Bn, and Si-Ov groups were significantly higher than in the millet monoculture (Si-Le). Additionally, the richness of bacterial communities ranked as follows: Si-Ts > Si-Bn > Si-Ov > Si-Le. The 20 samples analyzed were divided into four distinct groups, each corresponding to a specific rotation pattern. Significant differences among the groups were observed, with minimal variation within groups. This indicated good repeatability of the experimental data, and the differences between groups were greater than the intra-group differences.

**Figure 3:**
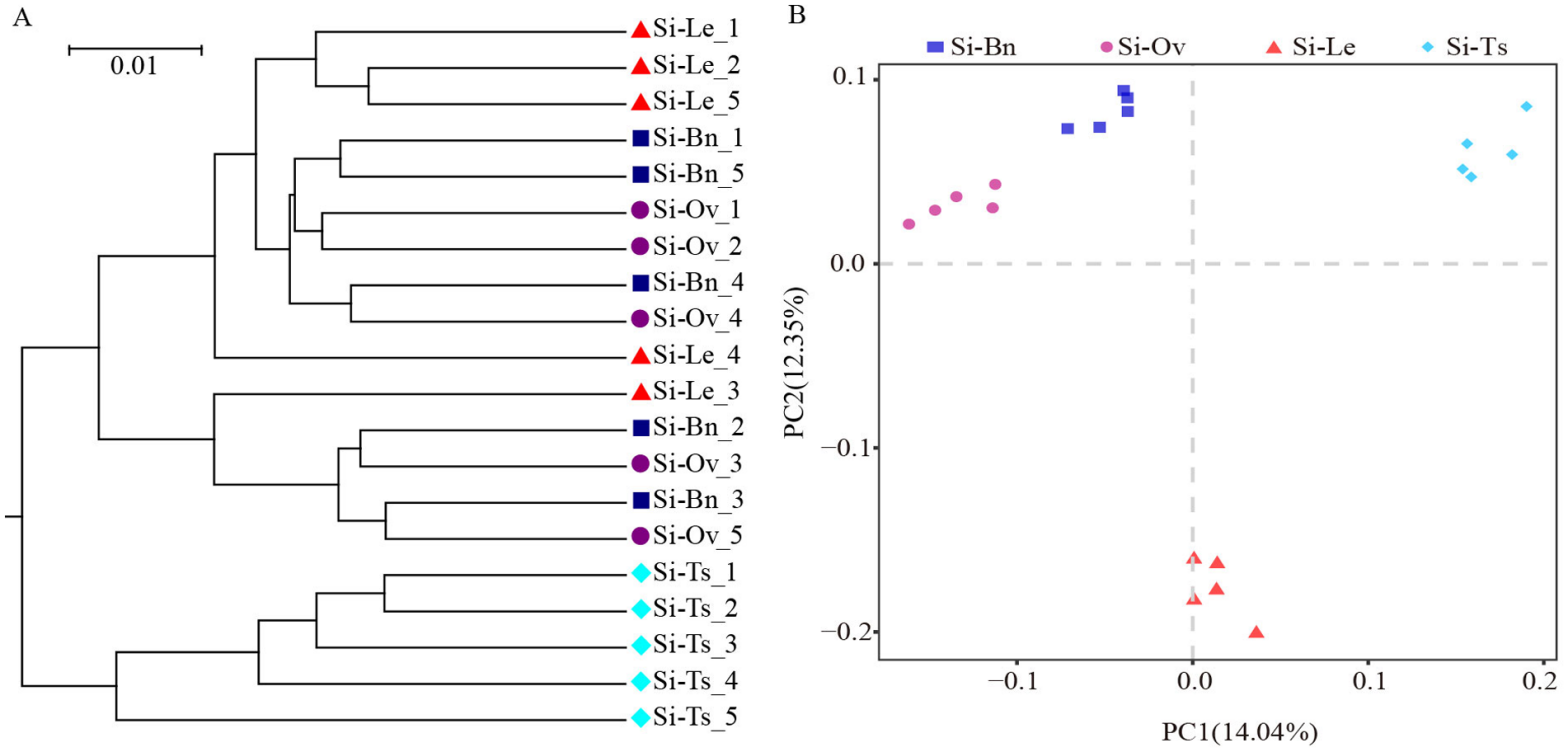
The hierarchical clustering and principal component analysis of bacterial communities. **(A)** Hierarchical clustering analysis of bacterial communities under different rotation patterns. Each color or shape represents a distinct sample or group, with branch lengths indicating relative distances between samples, reflecting their evolutionary similarity. **(B)** Principal Component Analysis (PCA) of beta diversity indices. The horizontal (PC1) and vertical (PC2) axes represent the primary factors influencing genetic composition variations among the sample groups. These axes are scaled to reflect relative distances without practical units and can be interpreted based on the characteristic features of the samples. The blue squares represent the soil samples of Si-Bn rotation pattern, The purple circles represent the soil samples of Si-Ov rotation pattern, The green rhomboids represent the soil samples of Si-Ts rotation pattern, The red triangles represent the soil samples of Si-Le rotation pattern.

Hierarchical clustering was employed to analyze the evolutionary distances of bacteria at the OTU level across different rotation patterns. The branch lengths in the evolutionary tree represent the distances between samples: shorter branch lengths indicate higher similarity between samples, which cluster more closely together. These results are illustrated in Figure 3A, where the Si-Ts group is distinctly separated from the other three groups. Phylogenetic diversity, based on distance spectrum diversity, reflects the similarity among samples. The closer the points on the plot, the higher the similarity between the samples. Two key conclusions can be drawn from this analysis: Differences in community composition among samples within the same group can be identified. Variations in community composition among samples across different groups can be assessed based on the distances between groups. PCA reflects the difference of multiple groups of data on the two-dimensional coordinate graph, and the coordinate axis can reflect the two eigenvalues of the variance value to the maximum. The difference and distance between samples can be reflected by analyzing the genetic composition of different samples. The more similar the composition of the samples, the closer the distance reflected in the PCA diagram. The soil samples in the same crop rotation pattern are clustered together, and the soil samples in different crop rotation patterns have a certain distance, reflecting the repeatability of the samples in the same rotation pattern and the differences between the samples in different crop rotation patterns (Figure 3B).

### 3.4 Analysis of bacterial community composition

**Figure 4:**
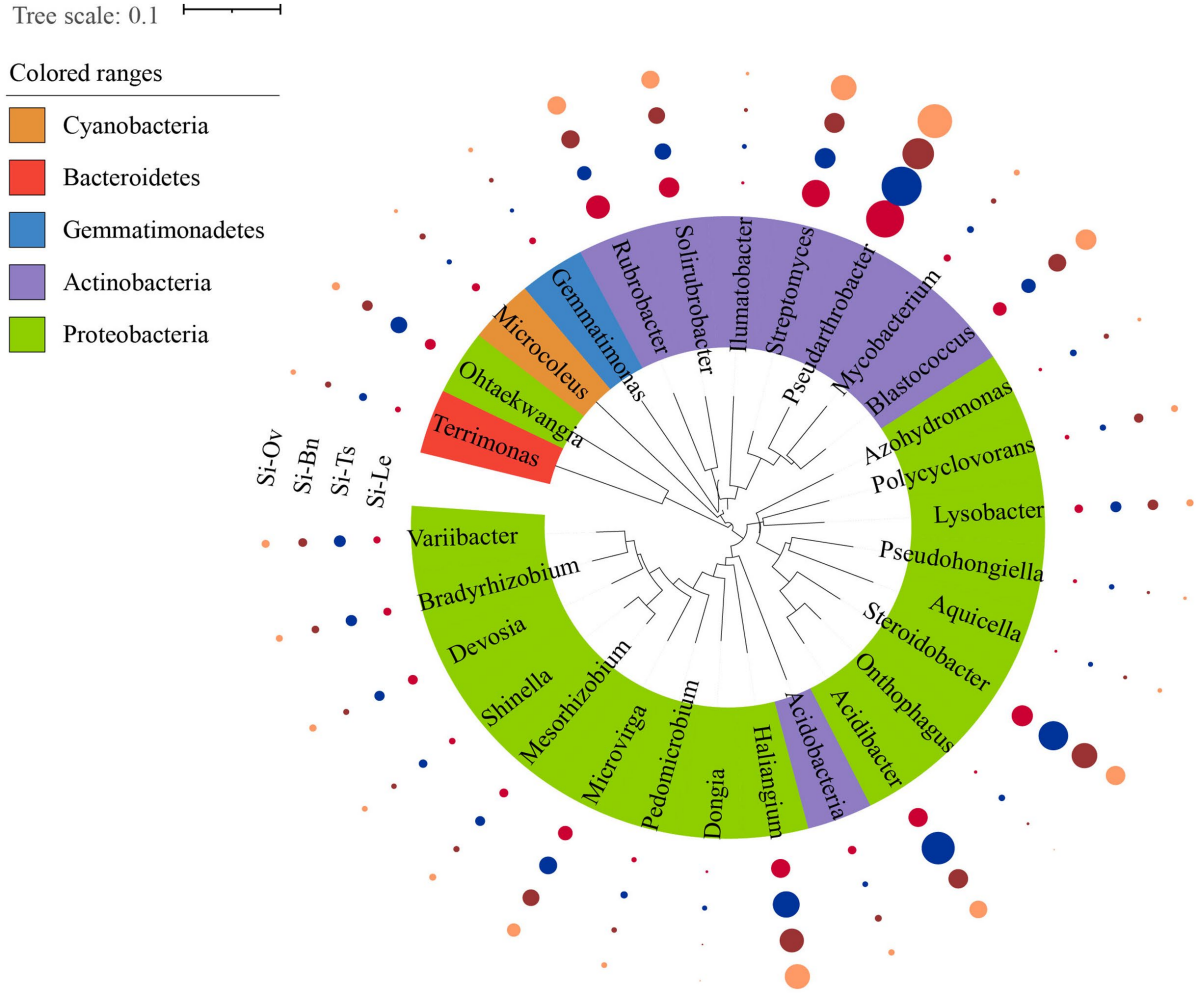
Differential analysis of bacterial genera across rotation patterns. Colored circles represent the relative abundance of each genus. The higher the significant difference, the bigger the circle’s diameter. Taxonomic dendrogram shows the inferred evolutionary relationship of the enriched microbiota of each sample. The total relative abundances of all genera and their significant effects across soil compartments are detailed in Table 3.

High-throughput sequencing was conducted to analyze the composition of soil rhizosphere bacterial community. The analysis results revealed that: The bacterial communities cross all samples were mainly composed of Proteobacteria, Actinobacteria, Acidobacteria, Chloroflexi, and Bacteroidetes. Among the groups, the proportion of Proteobacteria was highest in the Si-Ts group (30.2%), followed by the Si-Bn (26.7%), Si-Ov (25.8%), and Si-Le (25.2%) groups. The second most abundant phylum was Actinomycetes, accounting for 20.7 to 23.1% across the groups. The variance analysis of the top 60 genera in terms of relative abundance identified 29 genera with significant differences in different rotation patterns. These included: 6 OTUs belong to Proteobacteria, such as Microvirga, Bradyrhizobium, Devosia, Streptomyces, Steroidobacter, Dongia, Azohydromonas and Shinella granulosa; 8 OTUs belong to actinomycetes, such as Pseudarthrobacter, Blastococcus, Solirubrobacter and Rubrobacter. Streptococcus (Ohtaekwangia) and Terrimonas belonged to bacteroidetes were showed in Figure 4.

Compared with Si-Le group, the relative proportions of *Pseudohongiella* in Si-Ts, Si-Bn and Si-Ov groups were increased by 12.8, 13.8, and 1.25%, respectively. The relative proportion of *Microvirga* in Si-Ts and Si-Bn group was 30.6% and 15.2% higher than that in Si-Le group, while the relative proportion of *Microvirga* in Si-Ov group was 19.6% lower than that in Si-Le group. The relative proportions of *Bradyrhizobium* and *Mesorhizobium* in Si-Ts group were 41.9 and 33.2% higher than those in Si-Le group. While the relative proportion of *Bradyrhizobium* in Si-Bn and Si-Ov groups was significantly reduced by 5.1 and 14.0% compared with that in Si-Le group. The relative proportion of *Mesorhizobium* in Si-Bn and Si-Ov groups was significantly decreased, the reduction ratio was 23.6 and 21.1% compared with that in Si-Le group. The relative proportions of *Devosia* in Si-Ts, Si-Bn and Si-Ov groups were 61.6, 1.7 and 18.5% higher than those in Si-Le group. The relative proportion of *Shinella granules* in Si-Ts group was significantly increased by 30.0% compared with Si-Le group, while the relative proportion in Si-Bn and Si-Ov group was significantly decreased by 23.7 and 21.7% compared with Si-Le group. The relative proportions of *Azohydromonas* in Si-Ts, Si-Bn, and Si-Ov groups were 92.1, 25.0, and 138.4% higher than those in Si-Le group, respectively. The relative proportion of *Pseudomonas* (*Dongia*) in Si-Ts group was significantly increased by 81.3% compared with Si-Le group, while the relative proportion in Si-Bn and Si-Ov group was significantly decreased by 27.5 and 47.6% compared with Si-Le group. The relative proportions of *Haliangium* in Si-Ts, Si-Bn and Si-Ov groups were 63.4, 22.0, and 62.7% higher than those in Si-Le group. The relative proportion of *Lysobacter* in Si-Ts and Si-Bn group was 48.9 and 47.3% higher than that in Si-Le group. The relative ratio of *Steroidobacter* in Si-Ts group was significantly increased by 10.6% compared with Si-Le group, while the relative ratio in Si-Bn and Si-Ov group was significantly decreased by 34.4 and 16.4% compared with Si-Le group. The proportion of *Ohtaekwangia* in Si-Ts group was significantly increased by 158% compared with Si-Le group, but the proportion of *Ohtaekwangia* in Si-Bn and Si-Ov group was significantly decreased by 22.9 and 11.3% compared with Si-Le group. The relative ratio of *Ilumatobacter* in Si-Ts, Si-Bn, and Si-Ov groups was increased by 24.0, 12.1, and 51.3% compared with Si-Le group, respectively. The relative proportions of *Streptomyces* in Si-Ts, Si-Bn, and Si-Ov groups were 18.8, 43.5, and 21.9% higher than that in Si-Le group (Table 3).

**Table 3:** The proportion of enriched differential OTUs in every group.

ID	Si-Le	Si-Bn	Si-Ov	Si-Ts	Phylum	Order	Class	Family	Genus
OTU_3577	0.0157	0.0131	0.0143	0.0167	Actinobacteria	Micrococcales	Actinobacteria	Micrococcaceae	*Pseudarthrobacter*
OTU_3502	0.0057	0.0074	0.0087	0.0060	Actinobacteria	Frankiales	Actinobacteria	Geodermatophilaceae	*Blastococcus*
OTU_4813	0.0045	0.0052	0.0036	0.0059	Proteobacteria	Rhizobiales	Alphaproteobacteria	Methylobacteriaceae	*Microvirga*
OTU_4678	0.0033	0.0031	0.0028	0.0047	Proteobacteria	Rhizobiales	Alphaproteobacteria	Bradyrhizobiaceae	*Bradyrhizobium*
OTU_4819	0.0033	0.0022	0.0026	0.0036	Proteobacteria	Rhizobiales	Alphaproteobacteria	Hyphomicrobiaceae	*Devosia*
OTU_4816	0.0027	0.0022	0.0024	0.0035	Proteobacteria	Rhizobiales	Alphaproteobacteria	Rhizobiaceae	*Shinella*
OTU_4825	0.0022	0.0017	0.0017	0.0029	Proteobacteria	Rhizobiales	Alphaproteobacteria	Phyllobacteriaceae	*Mesorhizobium*
OTU_868	0.0013	0.0011	0.0006	0.0027	Proteobacteria	Xanthomonadales	Gammaproteobacteria	uncultured	*Bacterium_endosymbiont_of_Onthophagus_Taurus*
OTU_810	0.0039	0.0030	0.0032	0.0027	Actinobacteria	Solirubrobacterales	Thermoleophilia	Solirubrobacteraceae	*Solirubrobacter*
OTU_856	0.0010	0.0019	0.0012	0.0024	Proteobacteria	Burkholderiales	Betaproteobacteria	Comamonadaceae	*Azohydromonas*
OTU_87	0.0035	0.0028	0.0026	0.0023	Acidobacteria	Acidobacteria_bacterium_WX27	Subgroup_6	Acidobacteria_bacterium_WX27	*Acidobacteria_bacterium_WX27*
OTU_4172	0.0010	0.0016	0.0013	0.0021	Proteobacteria	Rhizobiales	Rhizobiales	Xanthobacteraceae	*Variibacter*
OTU_1668	0.0030	0.0030	0.0027	0.0020	Actinobacteria	Rubrobacterales	Rubrobacteria	Rubrobacteriaceae	*Rubrobacter*
OTU_4159	0.0013	0.0016	0.0015	0.0020	Actinobacteria	Acidimicrobiales	Acidimicrobiales	Acidimicrobiaceae	*Ilumatobacter*
OTU_4827	0.0010	0.0007	0.0005	0.0018	Proteobacteria	Rhodospirillales	Rhodospirillales	Rhodospirillaceae	*Dongia*
OTU_3641	0.0013	0.0023	0.0015	0.0018	Cyanobacteria	SubsectionIII	Cyanobacteria	FamilyI	*Microcoleus*
OTU_1725	0.0011	0.0018	0.0013	0.0018	Proteobacteria	Myxococcales	Deltaproteobacteria	Haliangiaceae	*Haliangium*
OTU_2839	0.0006	0.0005	0.0005	0.0016	Bacteroidetes	Cytophagales	Cytophagales	Cytophagaceae	*Ohtaekwangia*
OTU_871	0.0010	0.0015	0.0008	0.0015	Proteobacteria	Xanthomonadales	Xanthomonadales	Xanthomonadaceae	*Lysobacter*
OTU_876	0.0008	0.0009	0.0008	0.0009	Proteobacteria	Oceanospirillales	Oceanospirillales	Oceanospirillaceae	*Pseudohongiella*
OTU_1054	0.0006	0.0004	0.0005	0.0007	Proteobacteria	Xanthomonadales	Xanthomonadales	Xanthomonadales_Incertae_Sedis	*Steroidobacter*
OTU_3546	0.0005	0.0006	0.0007	0.0006	Actinobacteria	Streptomycetales	Streptomycetales	Streptomycetaceae	*Streptomyces*
OTU_902	0.0001	0.0002	0.0002	0.0004	Proteobacteria	Legionellales	Legionellales	Coxiellaceae	*Aquicella*
OTU_3711	0.0004	0.0003	0.0004	0.0004	Actinobacteria	Corynebacteriales	Corynebacteriales	Mycobacteriaceae	*Mycobacterium*
OTU_3040	0.0004	0.0003	0.0003	0.0003	Gemmatimonadetes	Gemmatimonadales	Gemmatimonadales	Gemmatimonadaceae	*Gemmatimonas*
OTU_4868	0.0003	0.0003	0.0003	0.0003	Planctomycetes	Phycisphaerales	Phycisphaerales	Phycisphaeraceae	*SM1A02*
OTU_2559	0.0000	0.0000	0.0000	0.0002	Bacteroidetes	Sphingobacteriales	Sphingobacteriales	Chitinophagaceae	*Terrimonas*
OTU_5105	0.0000	0.0001	0.0001	0.0001	Proteobacteria	Rhizobiales	Rhizobiales	Hyphomicrobiaceae	*Pedomicrobium*
OTU_2000	0.0000	0.0001	0.0001	0.0000	Proteobacteria	Xanthomonadales	Xanthomonadales	Solimonadaceae	*Polycyclovorans*

### 3.5 Relationship between soil properties and bacterial community composition

**Figure 5:**
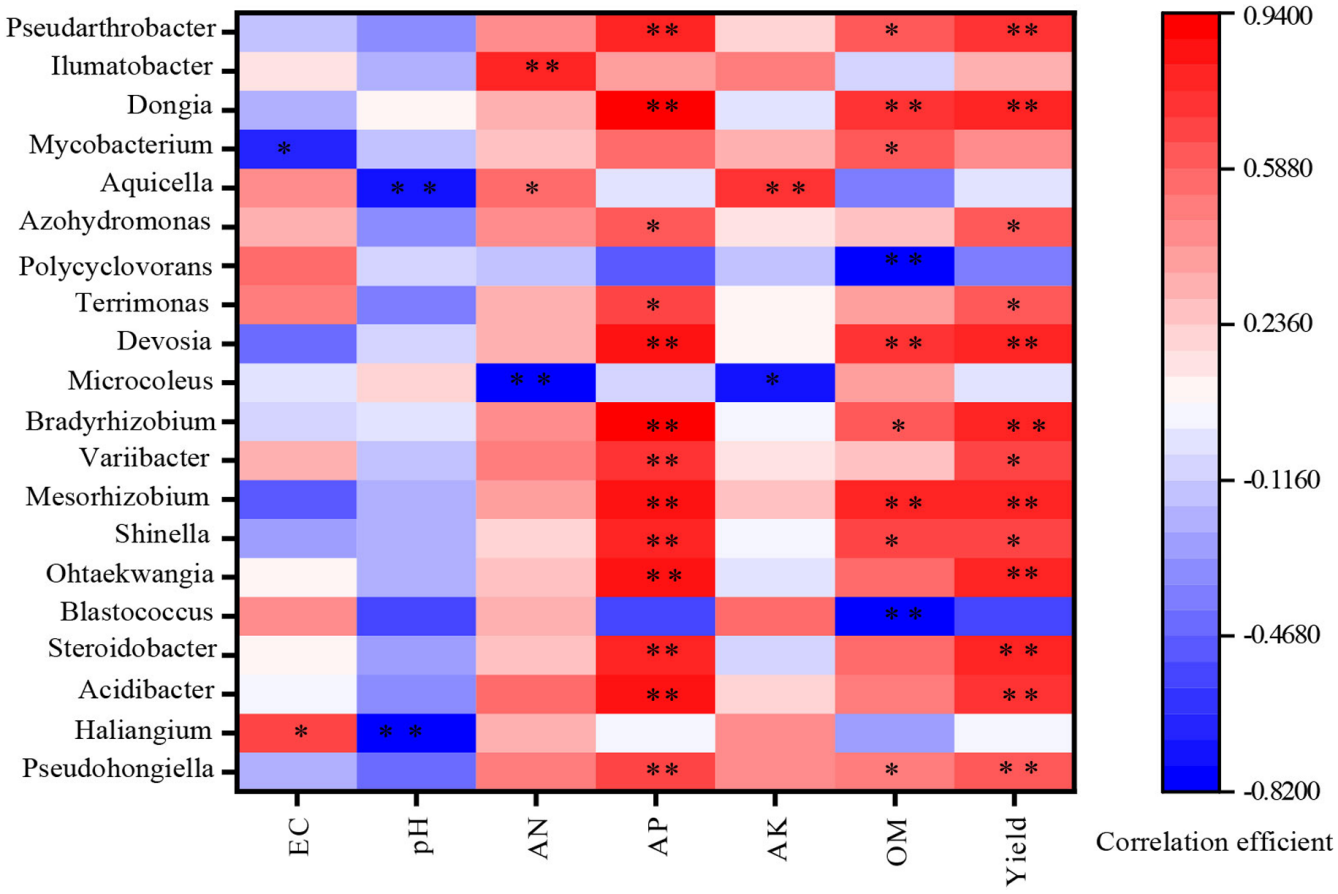
Pearson correlations between differential genus and physicochemical properties. pH, pH Value; EC, electric conductivity; OM, organic matter; AP, available potassium; AK, available phosphorus; AN, alkali-hydrolyzed nitrogen; Yield, grain yield. *p < 0.05, **p < 0.01.

To analyze the relationship between soil bacterial flora and soil physical and chemical properties across different rotation patterns, correlations were examined between environmental factors and 29 bacterial genera that showed significant differences among the patterns. The result revealed that each of the 29 bacterial genera was significantly correlated with at least one soil physicochemical property. *Pseudarthrobacter, Dongia*, *VosseliaDevosia*, *Bradyrhizobium*, *Mesorhizobium*, and *Shinella* were significantly positively correlated with available phosphorus and organic matter in soil. *Azohydromonas*, *Terrimonas*, *Streptomyces*, *Steroidobacter*, and *Ohtaekwangia* were only significant positively correlated available phosphorus in soil. *Microcoleus* had a significant negative correlation with alkali nitrogen and available potassium of soil species. There was a significant negative correlation between *Haliangium* and soil pH. *Blastococcus* exhibited a significant negative correlation with soil organic matter. These relationships are illustrated in Figure 5 and summarized in Table 4.

**Table 4:** The Pearson correlation between differential genus and physicochemical parameters.

Genus name	EC	pH	AHN	AP	AK	OM	Yield
*Pseudarthrobacter*	−0.197	−0.451	0.504	0.703*	0.431	0.516	0.612*
*Haliangium*	0.649*	0–0.816**	0.318	0.036	0.448	−0.256	0.03
*Acidibacter*	0.057	−0.348	0.53	0.841**	0.217	0.525	0.762**
*Steroidobacter*	0.097	−0.291	0.268	0.804**	−0.091	0.531	0.790**
*Blastococcus*	0.428	−0.533	0.330	−0.556	0.555	−0.764**	−0.545
*Ohtaekwangia*	0.103	−0.213	0.292	0.875**	−0.006	0.539	0.821**
*Shinella*	−0.263	−0.199	0.231	0.784**	0.039	0.663*	0.656*
*Mesorhizobium*	−0.500	−0.178	0.400	0.830**	0.263	0.785**	0.774**
*Variibacter*	0.343	−0.161	0.487	0.739**	0.148	0.290	0.659*
*Bradyrhizobium*	−0.073	−0.045	0.439	0.911**	0.059	0.597*	0.822**
*Microcoleus*	−0.040	0.221	−0.765**	−0.064	−0.703*	0.378	−0.049
*Devosia*	−0.437	−0.068	0.351	0.861**	0.109	0.760**	0.767**
*Terrimonas*	0.472	−0.372	0.335	0.680*	0.106	0.38	0.629*
*Polycyclovorans*	0.548	−0.098	−0.127	−0.500	−0.126	−0.774**	−0.386
*Azohydromonas*	0.343	−0.345	0.460	0.597*	0.162	0.255	0.635*
*Aquicella*	0.442	−0.751**	0.584*	−0.027	0.720**	−0.373	−0.025
*Mycobacterium*	−0.686*	−0.139	0.282	0.541	0.298	0.604*	0.425
*Dongia*	−0.224	0.091	0.312	0.936**	−0.015	0.761**	0.806**
*Ilumatobacter*	0.164	−0.204	0.767**	0.364	0.511	−0.062	0.314
*Pseudohongiella*	−0.138	−0.339	0.420	0.796**	0.216	0.608*	0.713**

*p < 0.05, **p < 0.01.

**Figure 6:**
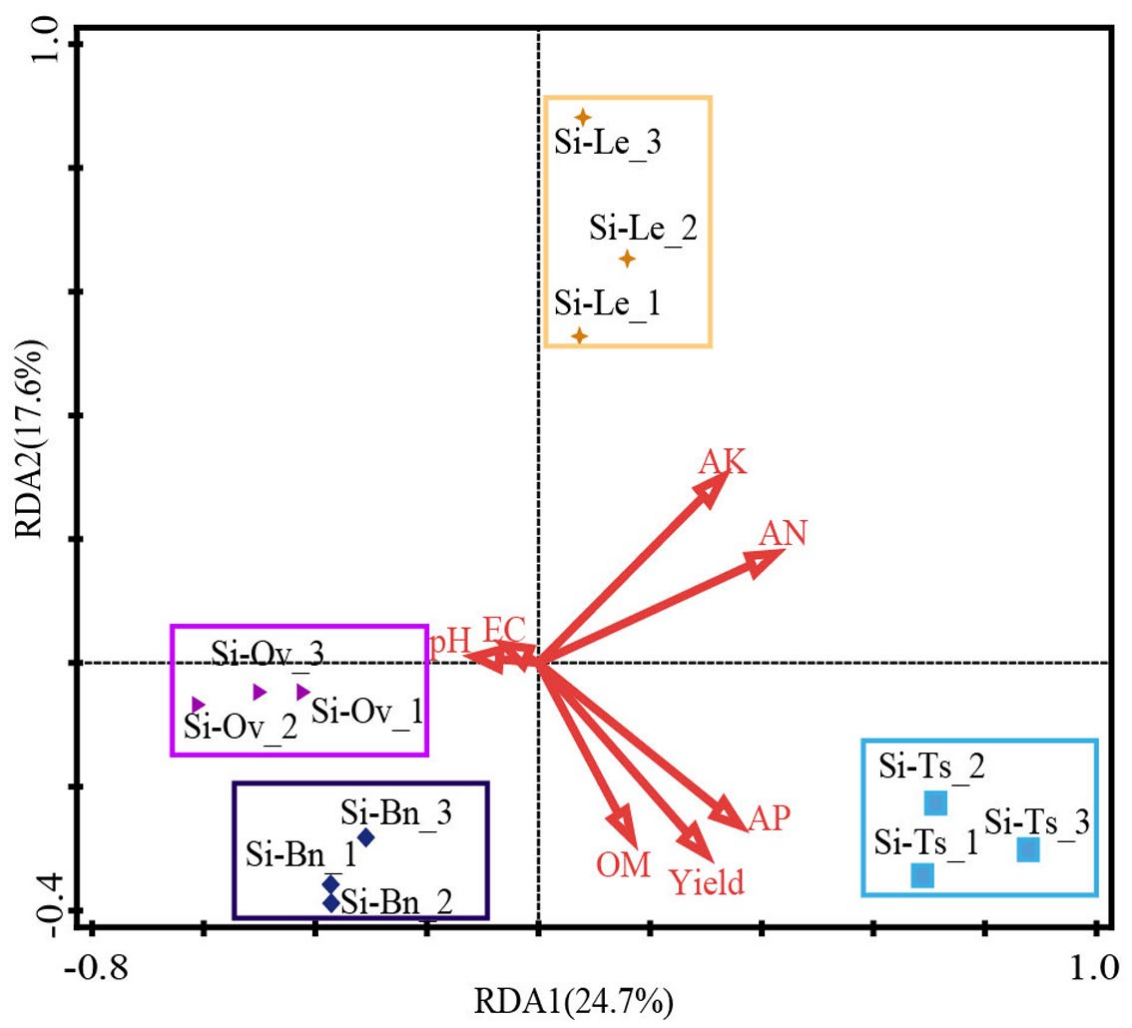
RDA of bacterial communities and environmental factor for individual samples. pH, pH Value; EC, electric conductivity; OM, organic matter; AP, available potassium; AK, available phosphorus; AN, alkali-hydrolyzed nitrogen; Yield, grain yield.

To evaluate the impact of environmental factors on soil bacterial community structure, redundancy analysis (RDA) was conducted to explore the relationships between these factors and the bacterial community. The analysis revealed that soil alkali-hydrolyzed nitrogen, available potassium, available phosphorus, and organic matter were positively correlated with the bacterial community structure across all samples, while pH and electrical conductivity (EC) were negatively correlated. These findings are illustrated in Figure 6. RDA1 and RDA2 accounted for 24.7 and 17.6% of the total variation, respectively. Among the individual environmental factors, alkali-hydrolyzed nitrogen contributed the most, explaining 21.6% of the variation with a contribution rate of 28.8%. Available phosphorus explained 18% of the variation, with a contribution rate of 23.9%. A significant correlation was observed between soil alkali-hydrolyzed nitrogen, available phosphorus, and bacterial community structure (Table 5). In summary, alkali-hydrolyzed nitrogen and available phosphorus were identified as the key factors driving structural changes in the soil bacterial community (*p* < 0.05).

**Table 5:** Forward selection results of RDA.

Name	Explains %	Contribution %	pseudo-F	*p*-value
AHN	21.6	28.8	2.8	0.004
AP	18	23.9	2.7	0.002
OM	8.6	11.4	1.3	0.092
pH	8.3	11.1	1.3	0.148
AK	7.6	10.1	1.3	0.228
Yield	5.6	7.5	0.9	0.548
EC	5.5	7.3	0.9	0.508

pH, pH value; EC, electric conductivity; OM, organic matter; AP, available potassium; AK, available phosphorus; AN, alkali-hydrolyzed nitrogen; Yield, grain yield.

## 4 Discussion

### 4.1 Effects of different rotation patterns on millet yield and yield components

Crop rotation has been shown to enhance millet yield (Li et al., 2020). This study, revealed that millet yield significantly increased under millet rotation systems involving *Triticum secale*, *Brassica napus L.*, and *Orychophragmus violaceus* compared to the fallow-millet system in 2021 and 2022. Among these, the *Triticum secale*-millet rotation showed the highest yield improvement. Although the millet yield were declined in 2022 compared to that in 2021, but the millet yields in different manure-millet rotation patterns were still higher than that in miller-fallow rotation pattern. This year-to-year variation may be attributed to climatic factors, including total precipitation, precipitation distribution, accumulated temperature, and sunlight duration (Xia et al., 2023). The fallow-millet rotation exhibited the most pronounced yield reduction in 2022, potentially due to continuous cropping issues. These issues often lead to the accumulation of pests and diseases, as well as shifts in the soil microbial community (Tian et al., 2017; Pavithran et al., 2022). Returning green manure to the soil has been reported to enhance crop yield components (Trivedi et al., 2016). Consistent with this, our study demonstrated that the *Triticum secale*-millet rotation significantly increased the 1,000-grain weight of millet, while the *Orychophragmus violaceus*-millet rotation significantly improved spike grain weight. Yield was found to be significantly positively correlated with 1,000-grain weight and grain weight per spike, aligning with findings from other studies (Qian et al., 2022). These results suggest that incorporating green manure crops like *Triticum secale*, *Brassica napus L.*, and *Orychophragmus violaceus* can improve important economic traits of millet, ultimately boosting overall yield.

### 4.2 Effects of different rotation patterns on soil physical and chemical properties

Effective irrigation and well-designed crop rotation systems are critical for enhancing soil nutrient profiles (Wang L. et al., 2022; Wang X. Y. et al., 2022). Crop rotation can increase the soil productivity (Wu et al., 2025). This study demonstrated that compared to the fallow-millet system, *Triticum secale*-millet and *Brassica napus* L.-millet rotations significantly increased the organic matter content in the topsoil. This can be attributed to the high carbohydrate content in *Triticum secale* and *Brassica napus L.*, which promotes the accumulation of organic matter while accelerating soil mineralization (Gao et al., 2021). These findings are consistent with previous studies (Li et al., 2019). Additionally, green manure crops have been shown to rejuvenate soil humus, enhance soil organic matter, and improve phosphorus availability (Zhao et al., 2019). Changes in the soil microbial community likely play a central role in improving the efficiency of soil nutrient utilization (Latkovic et al., 2020). The results of this study highlighted a significant increase in soil available phosphorus under the *Triticum secale*-millet rotation compared to the fallow-millet system. The RDA analysis further revealed a strong positive correlation between soil bacteria, alkali-hydrolyzed nitrogen, and available phosphorus. Moreover, available phosphorus was highly correlated with yield and specific bacterial taxa exhibiting significant differences across cropping patterns in Figure 4. These findings suggest that the *Triticum secale*-millet rotation enhances the microbial capacity to absorb and degrade phosphorus, thereby improving the availability of phosphorus and promoting millet yield.

### 4.3 Effects of different rotation patterns on soil bacterial community structure

Soil microorganisms play a crucial role in influencing plant productivity by establishing symbiotic, parasitic, or mutualistic relationships with plants (Van der heijden et al., 2008). Among these, bacteria are the dominant components of soil microorganisms, and their diversity and population composition are critical indicators of soil health and vitality (Amato et al., 2013). Research has demonstrated that crop rotation, as opposed to continuous cropping, enhances microbial diversity, alters population structures, and promotes biological activity (Detheridge et al., 2016; Chapatto et al., 2012). Greater microbial diversity enriches the soil environment, contributing to the stability and health of the soil ecosystem (Pietras et al., 2013; Wang et al., 2017). In this study, 16S RNA high-throughput sequencing was conducted to analyze the rhizosphere microbial communities of millet under four different cropping systems: *Triticum secale*-millet, *Brassica napus L.*-millet, *Orychophragmus violaceus*-millet, and millet monoculture. The results of alpha and beta diversity analyses revealed that different rotation patterns significantly enhanced rhizosphere bacterial diversity to varying extents. Among the cropping patterns, the diversity of soil rhizosphere bacteria ranked as follows: *Triticum secale*-millet rotation > *Brassica napus*-millet rotation > *Orychophragmus violaceus*-millet rotation > fallow-millet.

The degree of improvement in microbial community structure and composition varied depending on the crop rotation combination (Chaer et al., 2009). This study showed that rotation systems involving millet with other crops led to changes in soil bacterial composition. The relative abundances of *Proteobacteria* (e.g., *Pseudohongiella, Dongia, Azohydromonas, Microvirga, Bradyrhizobium, Mesorhizobium, Devosia, Haliangium, Lysobacter,* and *Steroidobacter*), *Bacteroidetes* (e.g., *Ohtaekwangia*), and *Actinobacteria* (e.g., *Ilumatobacter* and *Streptomyces*) were higher in the Triticum secale-millet, *Brassica napus*-millet, and *Orychophragmus violaceus*-millet rotation modes compared to millet monoculture. Among these, the Triticum secale-millet rotation exhibited the highest species richness and relative abundance. Correlation analysis further demonstrated a significant positive relationship between these bacterial taxa and millet yield. Previous studies have highlighted the role of *Pseudomonas* as a key rhizosphere-promoting microorganism with biocontrol functions, producing chitinase and lysozyme while enhancing plant nutrition (Ai et al., 2018; Gopalakrishnan et al., 2015). The increased availability of soil phosphorus (P) has been linked to higher bacterial diversity and abundance, potentially due to its role in adsorption and decomposition processes (Han et al., 2017). In this study, the relative abundance of several genera within *Bacteroidetes* and *Proteobacteria* was significantly higher in the rotation systems than in fallow-millet, while the relative abundance of *Acidobacteria* decreased by 19.1, 26.8, and 33.44% in the *Brassica napus*-millet, *Orychophragmus violaceus*-millet, and Triticum secale-millet rotations, respectively (Table 2).

Soil physical and chemical properties were positively correlated with the abundance of *Bacteroidetes* and *Proteobacteria*, but negatively correlated with *Acidobacteria*. Previous research has established that organic matter content is positively associated with *Bacteroidetes* and *Proteobacteria*, but negatively associated with *Acidobacteria* (Li et al., 2017). The findings of this study align with those observations, further underscoring the beneficial impact of crop rotation on soil bacterial communities and overall soil health.

## 5 Conclusion

The rotation of *Triticum secale*, *Brassica napus*, and millet proved effective in enhancing the accumulation of organic matter and available phosphorus in surface soil. These rotation systems significantly increased the abundance of beneficial bacterial communities in the rhizosphere, while reducing the prevalence of harmful bacterial populations. Additionally, they contributed to improved millet yield by enhancing yield components. Among the tested rotation modes, the *Triticum secale*-millet rotation demonstrated the greatest potential for enriching beneficial soil bacteria and maintaining stable and high millet yields, making it particularly suitable for the low plain areas of Hebei Province. This study will provide a theoretical basis for advancing crop rotation strategies and offer technical support for sustainable agricultural development.

## Data Availability

The datasets presented in this study can be found in online repositories. The names of the repository/repositories and accession number(s) can be found in the article/supplementary material.
